# Medical Legislation

**Published:** 1886-06

**Authors:** 


### Editorial.

---

### Medical Legislation.

During the last session of the Legislature, there was an unusual number of medical bills acted upon—some good, others bad, as, of course, must be the case. Among the many actively discussed was the bill to create a medical examining board. It was the one carefully prepared by the State Society, endorsed by the Erie County and many other medical societies. It provided for the appointment of a medical board by the regents of the University of the State, consisting of nine physicians. Three were to be selected by the State Society, three from the various colleges of the State, three from the two other schools of medicine.

Last year the bill was opposed by persons connected with some of the medical schools. This year the clause which was objectionable to them was omitted, and hence they offered little opposition. The physicians of the other schools of medicine and the proprietors of patent medicine establishments, who seem to think this measure was aimed at them, effectually killed the bill before it was fairly introduced, making an overwhelming and disastrous defeat for the friends of higher medical education. To review the history of this measure for several years, we are led to the belief that every concession has lessened its chances of success. While this State has been trying to legislate to protect itself against "legalized quacks," many States in the Union have passed such bills. We are confident that the original bill, as prepared by the committee of Erie County Medical Society, and emphatically endorsed by nearly every county society of the State, is the one which would stand the best chance of success, and one which we should again revive, introduce and push in the next session of the Legislature. This entirely divorces the teaching and licensing bodies. It is the bill in which the public have the most confidence. It is the bill which, sooner or later, will become a law in this State. Let us then return to our first and best measure. To us will belong the honor. To wait for the action of the State Society, as has been urged, is wholly unnecessary. It is the right of any number of citizens, or any society, to present to their member of the Legislature any measure which will benefit the masses. At the next meeting of the society this should be brought to the notice of its members, and immediate action urged.

Among the other bills was an amendment to the midwifery law of this county. This act has undoubtedly done good in this city and county, but there are some defects in the law which should have been corrected. There are two bills which are now in the hands of the Governor for signature. The first is the act to prevent the spread of contagious eye diseases in public institutions in our cities. It requires the examining of every child, before admission, to see if it be free from any form of contagious eye trouble; for the proper quarantining of children who suffer from such eye trouble, etc. If this bill becomes a law, it will tend to stamp out these most disastrous forms of disease, and do an untold amount of good. It is urgently demanded, and it is hoped the Governor will at once approve it.

The second one now in the hands of the Governor is a bill to allow the Board of Supervisors of this county to appropriate not more than $2,500 a year for the relief, or rather use of, the Buffalo Dispensary and the Women and Children's Dispensary of this city. When this bill was introduced into the Legislature, the public press, and all to whose notice the fact was brought, ridiculed the idea that it ever would become a law, but the surprising fact is before us that both houses of Legislature have passed this most objectionable act. In this city there are many—far too many—public dispensaries. Not less than eight are in full operation. They do some good, and the physicians who conduct them receive their reward in reputation and experience. They are well repaid, or they would never have established these dispensaries. In addition to being very objectionable special legislation, which is illegal, this kind of law benefits a few interested persons, and not the poor, as is represented. There are few, if any, physicians who do not do a vast amount of work which they know to be charitable. There are many who, in addition to such work, furnish medicine to the poor in their distress. There are many physicians who, day in and day out, attend the public hospitals and dispensaries of this city without reward or hope of pay. Is is right, then, that we should be taxed to pay for those who establish private hospitals or dispensaries? We would not protest against such legislation were it not for the fact that the poor of our city will receive just as good treatment if either one of these institutions should close its doors.

In the next place, these private dispensaries increase pauperism. Where the so-called poor are urged to come to be treated without compensation, many, able both to work and to pay, are cared for free of charge, thus encouraging the pauper spirit, already too prevalent. The dispensary which will do good to the poor, and, at the same time, keep from its doors this element, must investigate each and every case to see if it *is* needy. This is done in the Fitch Institute Dispensary in this city, and in the Buffalo Provident Dispensary, and many are the cases brought to notice which could easily pay a physician. Thus these patients are saved from pauperism. That this method of investigation is not practiced in the private dispensaries in this city is a well-known fact. Again, the method of charging a small fee, say from five to ten cents for the medicine furnished, has proved successful in a great many cities. In Buffalo it has not been tried to any extent, and no doubt would be pronounced a failure. If this were practiced here, the dispensaries, every one of them, could be made self-supporting, and there would be no need of invoking public aid. The Fitch Dispensary, the Buffalo Provident Dispensary, have every facility for caring for all classes of patients. Each department is under the care of a well-known specialist. To pay private individuals for performing work which is here cheerfully given for the asking to the worthy poor, is demanding far too much. Again, there is already one eye and ear dispensary here which receives aid from the county. This, with all the others, can certainly provide for the dependent classes. If they are not able so to do, there are thirteen city physicians whose duty it is to attend them. For these and many other reasons, we most earnestly protest against this act. Some say, allow this bill to become a law, and then oppose the appropriation before the Board of Supervisors. This places a dangerous power in the hands of the Board—a power which can be turned to political uses. Let us hope, then, that the Governor will veto this bill.

NOTE.—Since writing the above, we learn the Governor has signed the dispensary bill. It becomes now the duty of every physician to oppose the appropriation before the Board of Supervisors for the use of any dispensary.

